# Interaction between gut microbiota metabolites and dietary components in lipid metabolism and metabolic diseases

**DOI:** 10.1099/acmi.0.000403

**Published:** 2023-06-23

**Authors:** Til Bahadur Basnet, Srijana GC, Rajesh Basnet, Sadia Fatima, Mahpara Safdar, Bismillah Sehar, Ali Saad R. Alsubaie, Falak Zeb

**Affiliations:** ^1^​ Department of Epidemiology and Biostatistics, School of Public Health, Fujian Medical University, Fuzhou, PR China; ^2^​ Kanti Children’s Hospital, Kathmandu, Nepal; ^3^​ State Key Laboratory of Respiratory Disease, Guangzhou Institutes of Biomedicine and Health, Chinese Academy of Sciences, Guangzhou, PR China; ^4^​ Department of Biochemistry, Institute of Basic Medical Sciences, Khyber Medical University, Peshawar, Pakistan; ^5^​ Department of Environmental Design, Health and Nutritional Sciences, Allama Iqbal Open University, Islamabad, Pakistan; ^6^​ Department of Health and Social Sciences, University of Bedfordshire, Bedford, UK; ^7^​ Department of Public Health, College of Public Health, Imam Abdulrahman Bin Faisal University, Dammam, Saudi Arabia; ^8^​ Research Institute for Medical and Health Sciences, University of Sharjah, Sharjah, UAE

**Keywords:** gut microbiota, lipid metabolism, gut barrier, natural compound

## Abstract

Gut microbiota composition has caused perplexity in developing precision therapy to cure metabolic disorders. However, recent research has focused on using daily diet and natural bioactive compounds to correct gut microbiota dysbiosis and regulate host metabolism. Complex interactions between the gut microbiota and dietary compounds disrupt or integrate the gut barrier and lipid metabolism. In this review, we investigate the role of diet and bioactive natural compounds in gut microbiota dysbiosis and also the modulation of lipid metabolism by their metabolites. Recent studies have revealed that diet, natural compounds and phytochemicals impact significantly on lipid metabolism in animals and humans. These findings suggest that dietary components or natural bioactive compounds have a significant impact on microbial dysbiosis linked to metabolic diseases. The interaction between dietary components or natural bioactive compounds and gut microbiota metabolites can regulate lipid metabolism. Additionally, natural products can shape the gut microbiota and improve barrier integrity by interacting with gut metabolites and their precursors, even in unfavourable conditions, potentially contributing to the alignment of host physiology.

## Introduction

Human faeces contain over 3000 bacterial species, with most being commensal and some causing infectious diseases and associated metabolic disorders. Metabolic disorders such as cardiometabolic and liver diseases are becoming more significant concerns [1]. However, gut microbes contribute to food management by giving the host access to non-edible nutrients that are essential in digestion and physiology [2].

Consuming a diet rich in microbiota-accessible carbohydrates, with reduced processed food intake, and exposure to fermented food may be beneficial for preserving the gut microbiota. In addition, a diversified diet containing adequate carbohydrates, fibre, protein, fat, vitamins, minerals and natural compounds such as dietary polyphenols with antioxidant capacity can positively affect the gut microbiota.

Polyphenols and natural compounds further stimulate the generation of various metabolites in the gut that induce health benefits at physiological and cellular levels. The gut microbiota plays a significant role in regulating lipid metabolism, which can increase or decrease the risk of metabolic diseases. Microbiota-derived short-chain fatty acids (SCFAs) are linked with satiety and weight reduction, reduced inflammation, improved gut barrier function, and improved glucose and lipid metabolism [3].

The gut microflora can modify the host’s ability to digest lipids by altering energy harvest, storage and retention [4]. Metabolites produced by gut microbiota regulate host metabolism through physiological function and cellular pathways. For instance, gut microbes trigger the activation of stearoyl-CoA desaturase-1 and unsaturated fat elongase-5, leading to changes in the acyl-chain patterns of glycerophospholipids in monounsaturated and polyunsaturated fats. Furthermore, acetic acid, created by gut bacteria breaking down dietary fibre, is a building block for synthesizing C16 and C18 unsaturated fats in the liver [5].

## Microbiota diversity and metabolites

The core microbiome of the healthy human distal gut is diverse and complex, consisting of over 1000 species. Most are co-habiting species belonging to the phyla Firmicutes and Bacteroidetes, followed by Actinobacteria*,* Verrucomicrobia and Proteobacteria [6]. Ouwerkerk *et al*. proposed two unmistakable microbial biological systems in the intestinal system; the luminal and the mucosal microbiota [7]. More than 90 % of microscopic organisms in the luminal segment are Firmicutes and Bacteroidetes*,* with Actinobacteria*,* Proteobacteria and Verrucomicrobia comprising the minor phyla. Firmicutes are generally more prevalent in overflow than Bacteroidetes in individuals [8]. In contrast, the number and variety of microscopic organisms are often smaller in the mucosal layer [7].

**Fig. 1.**
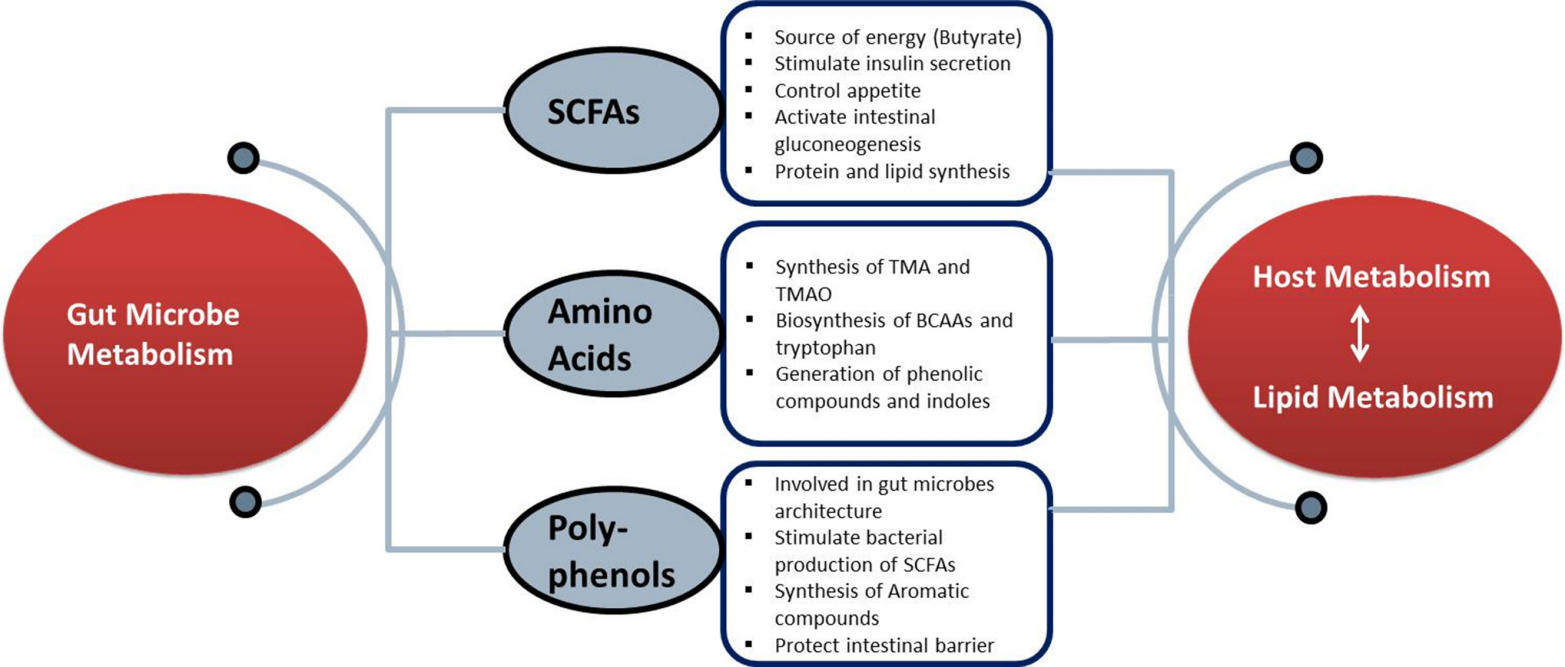
Gut microbiota metabolites that target lipid metabolism. Gut microbiota produces various metabolites (SCFAs, AAs, polyphenols etc) that execute many physiological and cellular functions for the regulation and targeting of lipid metabolism that ultimately target the body host metabolism.

Extensive evidence has demonstrated that the gut microbiota is involved in several diseases via distant dietary components, producing essential nutrients, minerals and metabolites [9–11]. The bidirectional relationship between the gut microbiota and microbe-derived components, including SCFAs, amino acids (AAs) and polyphenols may regulate physiological and molecular signals that maintain lipid metabolism (Fig. 1).

The primary microbiota metabolites include SCFAs, methylamines, AAs, polyphenols, indole subordinates, bile acids and benzoic acid [12]. These bacterial metabolites are released in the gut, and are later used by gut mucosal cells or assimilated in the flow and moved to the liver for transformation [13]. The bioactive molecules are generated because of the activities of the gut microbiota, which play a significant role in metabolic homeostasis, immunological cycles and neurobiological functions that are of essential significance in human health and disease [10, 11]. For example, some microbiota species produce SCFAs that serve as an energy source and regulate insulin production and satiate hunger by interacting with free fatty acid 2 and 3 receptors and releasing peptide hormones [14].

Butyrate, acetate and propionate are the most prevalent SCFAs generated by anaerobic gut bacteria, mainly through the fermentation of dietary fibre in the large intestine [15]. Locally, butyrate serves as the primary energy source for stomach mucosal cells. At the same time, propionate can be transported via the bloodstream to the liver, stimulating gluconeogenesis and increasing lipid synthesis and protein production. SCFAs also act as signalling molecules, influencing various physiological processes in the body, such as promoting gut barrier function and having anti-inflammatory effects [16]. Trimethylamine (TMA), an amine that is principally created from the diet, for example, l-carnitine, lecithin, choline and betaine, is oxidized by microbial catalysts into trimethylamine-N-oxide (TMAO) in the liver, fundamentally by the protein flavin-containing monooxygenase 3 (FMO3) [17].

*Enterobacteriaceae *, predominant gut microbes, produce a pool of circulating TMA, which can be converted back to TMAO in the liver by FMO3 [18]. The bacterial species *Prevotella copri* helps to confirm the biosynthesis of compounds, such as branched-chain amino acids and tryptophan. Therefore, it is noteworthy that these metabolites have a beneficial relationship with changes in the composition of the gut microbiome [19]. The most important compounds involved in the gut microbiota are polyphenols that engineer and invigorate bacterial creation of SCFAs [20]. Many intestinal bacteria, mostly Firmicutes*,* Bacteroidetes*,* Actinobacteria and Fusobacteria*,* can generate phenolic compounds and indoles from dietary amino acids [21]. Some colonic microbiota can synthesize benzoic corrosive, a fragrant carboxylic corrosive in the stomach, by ageing sweet-smelling dietary compounds [22].

## Crosstalk between gut microbiota and bioactive natural/chemical compounds in lipid metabolism

An increasing number of research investigations have been carried out to examine the beneficial impact of natural, chemical and bacterial substances [23] (Table 1) on microbiota dysbiosis. The impact of anti-microbials and probiotics on the gut microflora is associated with lipid metabolism and metabolic disorder in mice models [24]. For instance, natural plant-derived compounds, such as those found in *Coreopsis tinctoria* (CT), have been shown to modify blood lipid metabolism by reducing LDL levels without compromising liver function in both hyperlipidemic individuals and animal models. However, oral CT treatment significantly restored the gut’s microbial richness in mice with hyperlipidaemia [25]. In hyperlipidaemic rats, the levels of *Enterobacter* spp*., Lactobacillus* spp*.* and *Bifidobacterium* spp. were negatively correlated with cholesterol, fatty substances and lipoprotein, whereas certain bacterial flora, such as those containing *Clostridium leptum *, increased blood cholesterol, fatty acid levels and low-density lipoprotein levels [26].

**Table 1.** Effect of bioactive compounds on gut microbiota diversity and lipid metabolism. na, Not Applicable

Compound	Type	Study model	Effect on gut microbiota richness and diversity	Effect on lipid metabolism
*Coreopsis tinctoria*	Natural compound from plant	Hyperlipidaemic mice	Reversed microbiota richness and diversity	Decreased LDL
Xylitol	Chemical compound	High-fat experimental mice	Increased Firmicutes and *Prevotella *, decreased *Bacteroides* and *Barnesiella *	na
95 % ethanol extract (SPL95)	Extract from microalgae *Spirulina platensis*	High-fat diet rats	An abundance of beneficial bacteria (* Prevotella *, *Alloprevotella, Porphyromonadaceae, Barnesiella* and *Paraprevotella *)	Decreased lipid levels (serum TG, TC and LDL levels)
Medium- and long-chain triacylglycerols (MLCT) with 30 % (w/w) MCFA	Chemical compound	High-fat diet rats	Increase Firmicutes to *Bacteroidetes *	Decreased body weight gain and maintained serum lipid parameters and liver triacylglycerol content
sn2PA fat in combination with DHA or ARA	Chemical compound	Mice	Increased *Lactobacillus* in the faeces and decreased *Desulfovibrio *	Decreased liver triacylglyceride, increased total SCFA
Genistein	Natural compound	Dams	Increase *Bacteroides* and *Akkermansia *	Lower serum levels of TG and TC
Melatonin	na	Mice and human	An abundance of *Bacteroides* and *Alistipes* in animals and humans	Improved the production of acetic acid
Flavonoids	na	Mice and humans	Higher levels of *Bifidobacterium* spp. and *Bacteroides–Prevotella–Poryphyromonas,* but significantly lower levels of *Lactobacillus* spp.	Alleviated the adverse effects of metabolic diseases
Mono-2-ethylhexyl ester	Endocrine disruptor chemicals	na	Abundance of Firmicutes and reduction of *Verrucomicrobia *	na

ARA, arachidonic acid; DHA, docosahexaenoic acid; LDL, low-density lipoprotein; SCFA, short-chain fatty acid; sn2PA, Sn-2 palmitic acid triacylglycerols; TC, total cholesterol; TG, triglycerides.

Xylitol consumption decreased the phylum *Bacteroidetes* and the genus *Barnesiella* but increased the phylum Firmicutes and the genus *Prevotella* in both normal and high-fat mice [27]. A 95 % ethanol concentrate of SPL95 from microalgae *Spirulina platensis* enhanced beneficial micro-organisms, including *Prevotella *, *Alloprevotella *, *Porphyromonadaceae *, *Barnesiella* and *Paraprevotella *, while reducing the number of organisms such as *Turicibacter *, *Romboutsia *, *Phascolarctobacterium *, *Olsenella* and *Clostridium XVIII* connected with high levels of serum fatty oil, total cholesterol and low-density lipoprotein cholesterol levels [28]. Thus, the unsaturated fat from SPL95 can be used as an adjuvant treatment in food to improve gut microbiota in overweight and diabetic individuals.

A study found that consuming medium- and long-chain triacylglycerols (MLCT) with 30 % (w/w) MCFA reduced weight gain and maintained serum lipid levels and liver triacylglycerol content. Increased expression levels of genes encoding proteins for fat breakdown and decreased expression levels of genes encoding enzymes for fat synthesis were observed [29]. Additionally, a high-fat diet containing MLCT reduced the proportion of Firmicutes to Bacteroidetes and decreased the overall presence of Proteobacteria, which may be related to weight loss [29].

Sn-2 palmitic acid triacylglycerols mixed with DHA or ARA diminish fatty liver substance (TG) and increases the growth of *Lactobacillus* and SCFA concentration in the faeces while suppressing the growth of *Desulfovibrio *, demonstrating a beneficial impact on gastrointestinal well-being [30]. Genistein increased the abundance of beneficial gut bacteria, including *Bacteroides* and *Akkermansia *. It decreased TG and TC serum levels, suggesting high genistein intake could improve glucose homeostasis and insulin sensitivity [31]. Butyrate can alleviate microbiome dysbiosis caused by a methionine-choline deficient diet by reducing the levels of harmful bacteria like *Bilophila* and *Rikenellaceae* and improve beneficial probiotic genera like *Akkermansia, Roseburia, Coprococcus, Coprobacillus, Delftia, Sutterella,* and *Coriobacteriaceae *. The metabolites associated with lipid absorption and various pathways include arachidonic acid, stearic acid, oleic acid, linoleic acid, and squalene [32]

A study found that oral exposure to penicillin G (Pen G), erythromycin (Ery), or their combination increased lipid accumulation and essential characteristics related to fatty acid metabolism in the liver and modified the microbiota in the faeces and cecum. Using these antibiotics orally, especially in combination, could lead to microbiota dysbiosis, potentially causing metabolic issues and discomfort [33]. Another study showed that treatment with carbamazepine increased the relative abundance of Firmicutes*,* Proteobacteria and Actinobacteria, while Bacteroidetes declined in mice [34].

Despite its largely unknown mechanism, melatonin has been found to significantly affect the gut microbiota. Studies have shown that melatonin can improve gut microbiota populations, mainly by reducing the overgrowth of *Bacteroides* and *Alistipes* in animals and humans while enhancing lipid metabolism [35]. In mice fed a high-fat diet, melatonin treatment improved the production of SCFAs, which was associated with increased levels of *Bacteroides* and *Alistipes* [35]. Flavonoids have a dual effect on gut microbiota. On the one hand, prenylated isoflavonoids have antimicrobial activity against *Listeria monocytogenes* and methicillin-resistant *Staphylococcus aureus *, with prenylation at the β position being more predictive of antimicrobial action [36]. On the other hand, flavonoids can also increase the prevalence of certain bacteria in the gut, such as *Bifidobacterium* species in mice that consume a diet high in apple flavonoids. However, a concentrated population of *Bacteroides–Prevotella–Porphyromonas* bacteria can reduce the levels of *Lactobacillus* species in the gut [37]. Non-absorbable proanthocyanidins can alleviate metabolic disorders by acting on multiple organs, including the liver, pancreas, adipose tissue and brain, as well as directly in the gastrointestinal tract. These compounds can improve gut microbiota and gut barrier function and reduce inflammation [38].

Prebiotics such as inulin-type fructans, including fructooligosaccharides, oligofructose, inulin galactooligosaccharides and lactulose, promote the growth of beneficial micro-organisms, mainly unsaturated SCFA producers, *Bifidobacteria* and *Lactobacilli*, which have health-promoting activities [39, 40]. Other prebiotics such as resistant starches, trans-oligosaccharides, or xylooligosaccharides, lactosucrose, adhesives, chitin-glucans, and resistant dextrins are also being studied for their potential health benefits [39].

Ingestion of mono-2-ethylhexyl ester can cause a significant shift in microbiota composition, including an increase in Firmicutes and a decrease in Verrucomicrobia*,* according to a recent study on endocrine disruption. High-fat diets during the pubertal phase in male mice can lead to adipocyte breakage, which causes dyslipidaemia [41]. A few drugs, such as benzbromarone and allopurinol, can increase the abundance of beneficial bacteria, including *Collinsella* and *Bifidobacterium *, while decreasing the abundance of harmful bacteria, such as *Adlercreutzia* and *Anaerostipes *. Moreover, benzbromarone has been found to correct issues related to fat digestion in the gut microbiota of male rats with hyperuricaemia [42].

## The interplay between nutrition and microbiota in lipid metabolism

Dietary components impact significantly on the gut microbiota, with a high-fat diet leading to dysbiosis and lipid accumulation. A high-fat diet can increase harmful bacteria such as Firmicutes and Bacteroidetes while reducing beneficial bacteria such as *Akkermansia* and butyrate-producing bacteria, such as *Anaerotruncus *, *Butyricicoccus* and *Lactobacillus *. The mice that were fed with beef protein had impaired glucose digestion and insulin resistance and increased inflammatory markers (IL-1, TNF-α, IL-6, leptin, fatty substances, LDL cholesterol and total cholesterol) [35, 43]. Using essential oils in piglets increased the abundance of some bacterial genera (* Bacilli* and *Lactobacillales *, *Streptococcaceae* and *Veillonellaceae *) [44]. A combination of vitamin B12 and polyunsaturated fats (omega 3 fatty acids) prompted higher growth of the Gram-positive bacterium *S. aureus *. It diminished the endurance paces of *Clostridia* sp. and other intestinal bacterial strains [45]. The study indicates that dietary fats and oils from different sources cause dysbiosis in the gut microbiota.

## Gut nutrition and gut barrier: two sides of the same coin

The digestive monolayer is mainly composed of gastrointestinal epithelial cells and has various functions, such as regulating paracellular transport, transporting transcellular immunoglobulins and absorbing nutrients. Different types of cells within this layer, such as Challis, Paneth and resistant cells, perform specialized roles in defence against pathogens and maintaining a healthy gut environment [46]. The host-derived glycans, which include the cell surface glycocalyx and extracellular bodily fluid, form a thin layer that separates the mucosal microbiota from epithelial cells, playing a crucial role in human health and physiology. The layer is maintained by differences in composition between the mucosal microbiota and luminal content, and it is essential for nutrient exchange, communication with the host, immune system development and protection against invading micro-organisms [7]. The gut microbiota influences digestive barrier capabilities through microbial activity, inflammatory cytokines, endocannabinoids, nutrition, exercise and gastrointestinal peptides [47]. The microbiome maintains the gut epithelial obstruction’s dependability, protecting against the colonization of harmful micro-organisms, which is essential for gut homeostasis and functionality [48].

The endocannabinoid (eCB) framework proposed by Cani *et al*. is made of various bioactive lipids of N-acylethanolamines and acylglycerol families. The best portrayed are anandamide or N-arachidonoylethanolamine (AEA) and 2-arachidonoylglycerol (2-AG), which activate G-coupled cannabinoid receptors (CBR), in particular CB1R and CB2R [49]. During weight gain and diabetes, the gastrointestinal eCB system is modified, with an expanded overflow of AEA triggering stomach permeability through CB1R-dependent mechanisms [50]. This modification has been associated with the gut microbiota [49, 51]. Some gut microbacteria, such as *Akkermansia muciniphila *, can increase the levels of bioactive lipids like 2-oleoylglycerol (2-OG), 2-arachidonoylglycerol (2-AG) and 2-palmitoylglycerol (2 PG) [52] in the intestine.

Interestingly, the bioactive lipids, including 2-AG, 2 PG, and 2-OG, have been found to reduce metabolic endotoxemia and inflammation [53]. On the other hand, ligands for receptors involved in the release of stomach peptides, such as glucagon-like peptide-1 (GLP-1) and glucagon-like peptide-2 (GLP-2), are two peptides that have been implicated in the control of glucose homeostasis and gut barrier function, respectively [54].

A diet deficient in fibre impairs the colonic inner mucus layer that typically separates bacteria from host cells [55]. Probiotics can help improve the gut microbiota’s health by modifying the gut bacterial community through gastrointestinal routes [56]. For instance, probiotic *Bacillus* can enhance intestinal mucosa shape, tight junctions and immune function by activating the TLR signalling system and increasing intestinal epithelial cell barrier and function [24]. A blended microbial consortium, such as the Mozzarella di Bufala Campana microbiota supplementation, has more protective effects against high-fat diet (HFD) initiated fat accumulation, fatty oil and cholesterol levels compared to single-strain probiotic supplementation, suggesting synergistic communications within the microbial consortium. Dietary micro-organisms with uncharacterized probiotic impurities play a significant role [57]. Altering the gut microbiota with prebiotic or probiotic feeding can reduce gastrointestinal discomfort, strengthen the integrity of the gut barrier, improve metabolic balance and speed up weight loss [58].

## Mechanistic approach of gut microbiota in metabolic diseases

The gut microbiota has various effects on human health and is believed to play a significant role in developing several diseases. Some metabolic disorders are linked to gut microbiota through lipid digestion and irritability components. Desmosomes, junctional protein complexes that provide a strong adhesive bond between adjacent host cells, can be affected by enteropathogenic *Escherichia coli* (EPEC), weakening cell bonds, and reduced blockage capacity of digestive epithelial cells. This damage is caused by the EPEC effector protein (EspH) and its inhibitory action on Rho GTPases [59]. Certain types of gut microbiota, such as *A. muciniphila* and *Bacteroides thetaiotaomicron *, have adapted to the glycan-rich environment by developing bodily fluid-corrupting chemicals and bodily fluid-restricting extracellular proteins [7].

The composition and numbers of gut microbiota have been found to vary depending on the body mass index (BMI), with increased levels of Firmicutes and reduced levels of Bacteroidetes at higher BMI, and the phylum Actinobacteria in particular significantly depleted at higher BMI [60]. Mice grown without germs in a research model were noticeably thinner than those raised usually [24]. Genetically obese mice had drastically different gut flora from their wild counterparts fed the same diet [25]. The transplantation of microbiota from lean and obese mice into germ-free mice resulted in a general increase in total body fat, especially in subjects transplanted with a microbiota from overweight individuals [18].

Furthermore, studies that transplanted adult human gut microbiota into microbe-free mice showed that weight-related bacterial aggregates could be relocated, suggesting that gut microbes play a significant role in determining body weight and fat storage [26, 27]. SCFAs provide energy from the gut microbiota and trigger significant signalling pathways responsible for glucose-induced insulin secretion from pancreatic β-cells. SCFAs are identified by a distinct subclass of nutrient-sensing G protein-coupled receptors (GPCRs) and two SCFA receptors (FFA2 and FFA3) [61]. On the other hand, SFA ligands activate l-cells in the colon to release oxyntomodulin, glucagon-like peptide-1 (GLP-1), pancreatic peptide YY3-36, and glucagon-like peptide-2 (GLP-2) [62], which are believed to increase satiety and eliminate excess glucose. Additionally, intestinal neurons and sympathetic ganglia in the stomach have been shown to activate the free fatty acid 3 receptor, increasing digestive gluconeogenesis and sympathetic outflow while decreasing hepatic glucose production and energy expenditure [61].

Obesity may be caused by metabolic endotoxaemia characterized by inflammation in adipocytes and adipose tissues, downregulation of certain genes and increased levels of lipopolysaccharides (LPS) [63]. Interestingly, a high-fat diet can prevent bacterial LPS from entering the bloodstream while at the same time activating the Toll-like receptor-4 signalling pathway. During the intensive stage of obesity induced by a high-fat diet, the Serpin A3N protein shows a significant increase in expression within the neuroendocrine nerve centre, likely due to unsaturated fat digestion [64].

*Polygonatum odoratum* extract has been found to modulate gut microbiota by producing SCFAs, which can reduce weight gain and improve dyslipidaemia [65]. Research has demonstrated that red wine polyphenols and procyanidins can have a positive impact on the micro-organisms in the gut by augmenting the number of beneficial bacteria, including *Bifidobacteria* and *Lactobacillus *, which are involved in protecting the digestive system. Moreover, these substances can encourage the growth of micro-organisms that produce butyrate, such as *Faecalibacterium prausnitzii* and *Roseburia *, while restricting the growth of harmful bacteria, such as *E. coli* and *Enterobacter cloacae *, which are known to produce LPS [66]. Blocking the eCB system can change the gut microbiota by increasing *A. muciniphila* and decreasing *Langnospiraceae* and *Erysipelotrichaceae *, which can reduce the risk of metabolic disease and obesity by adjusting the inflammatory mediators produced by macrophages [67]. Chronic stress has been linked to microbiota dysbiosis, which affects lipid metabolism and eCB generation, resulting in reduced neurogenesis. However, administering a *Lactobacillus* probiotic strain may reverse this pathological condition and be a potential therapeutic remedy for stress-associated depressive disorders [73].

In patients with type 2 diabetes, a decrease in common butyrate-delivering microbes and an increase in other uncommon micro-organisms was associated with gut microbial dysbiosis [68]. Germ-free animal studies suggest that a reduction in gut microbiota can impact on host metabolism and increase susceptibility to diabetes and obesity [69]. However, because of antibiotic use, clinical findings on the impact of microbial diversity on host metabolism in humans are inconclusive [70]. Supplementing diets with SCFAs can reduce obesity, improve insulin resistance and enhance metabolic profiles [71]. Selective modulation of the gut microbiota by two kefir strains, *Lactobacillus mali* and *L. kefiranofaciens,* might influence insulin sensitivity, GLP-1 production, barrier integrity and lipid metabolism [72]. Probiotics have often been shown to enhance metabolic profiles by modifying gut microbiota and treating type 2 diabetes by reducing glucose malfunction [73]. Indoleamine 2,3-dioxygenase constraint can improve tryptophan digestion and interleukin-22 production, which depend on the microbiota, leading to improved metabolic outcomes [74].

Consuming large amounts of choline, carnitine and phosphatidylcholine can lead to the formation of TMA by gut bacteria, which is then converted into TMAO by FMO3 and controlled by farnesoid X receptor signalling. Elevated concentrations of TMAO and its forerunners have been associated with an augmented risk of cardiovascular disease and mortality, irrespective of conventional risk factors [49]. However, separate studies have shown that elevated levels of TMAO, unrelated to l-carnitine therapy, can reduce aortic lesions in apolipoprotein E knockout animals with certain genetic mutations [75]. TMAO may have a complex role in cardiovascular health, which is not yet fully understood. Various factors, including diet and the influence of the gut microbiota and its metabolites, may be clinical biomarkers for assessing cardiovascular risk [76].

## Research questions and future perspective

The diversity and quantity of gut microbiota and their metabolites play a significant role in host metabolism, immunity and physiology. They have implications for obesity, diabetes, and cardiovascular and liver diseases. However, research on the association of microbiota with other diseases, such as autoimmune disease and cancer, is limited. Nonetheless, dietary interventions and natural products have demonstrated effects on regulating gut microbiota and lipid metabolism.

Researchers have studied the impact of natural and biological compounds in correcting gut microbiota dysbiosis, primarily in animal and human models. Changing the host’s diet can be critical in establishing a favourable gut microbiota–host relationship. Further systematic reviews are necessary to evaluate the efficacy of each compound in normalizing the gut microbiota and its association with host metabolism.

Despite studying natural compounds’ effects on maintaining gut barrier integrity, more evidence is needed to limit metabolic endotoxaemia and prevent metabolic disorders. Therefore, more diverse research is necessary to address concerns related to gut microbiota and its metabolites entirely. Some potential research viewpoints include identifying transcriptional factors acting as gut sensors and their target genes, explaining the dietary signalling pathways involved in gut microbiota activities, measuring the metabolic outcomes of gut metabolites on specific cells and organs, understanding the interactions between gut metabolite-related regulatory pathways and pro-inflammatory pathways, and identifying the genotypes in the gut microbiota that are responsible for targeting the particular genome involved in metabolic dysfunction.

## Conclusion

The gut microbiota is a critical environmental factor, and its metabolites can signal physiological processes in the host metabolism. Notably, the microbiota–host interface determines the environment for colonizing microbiota with a normal immune response. Any disturbance in the composition or abundance of bacterial cells, known as dysbiosis of the gut microbiota, can result in abnormal host physiology and disease.

The intricate correlation between dietary compounds and gut microbiota can disrupt or harmonize the gut barrier and lipid metabolism. Therefore, it is essential to examine how diet plans interact with the gut microbiota and its metabolites in metabolic disorders. Dietary components can improve the quality of the gut microbiota, producing bacterial metabolites that can modulate the immune system, lipid metabolism and host genetic signalling pathways. Natural products can modulate the gut microbiota through their antioxidant activities and nutrigenetic interactions in lipid metabolism.

## References

[1] Li J, Jia H, Cai X, Zhong H, MetaHIT Consortium (2014). An integrated catalog of reference genes in the human gut microbiome. Nat Biotechnol.

[2] Sharon G, Garg N, Debelius J, Knight R, Dorrestein PC (2014). Specialized metabolites from the microbiome in health and disease. Cell Metab.

[3] Morrison DJ, Preston T (2016). Formation of short chain fatty acids by the gut microbiota and their impact on human metabolism. Gut Microbes.

[4] Velagapudi VR, Hezaveh R, Reigstad CS, Gopalacharyulu P, Yetukuri L (2010). The gut microbiota modulates host energy and lipid metabolism in mice. J Lipid Res.

[5] Kindt A, Liebisch G, Clavel T, Haller D, Hörmannsperger G (2018). The gut microbiota promotes hepatic fatty acid desaturation and elongation in mice. Nat Commun.

[6] Hollister EB, Gao C, Versalovic J (2014). Compositional and functional features of the gastrointestinal microbiome and their effects on human health. Gastroenterology.

[7] Ouwerkerk JP, de Vos WM, Belzer C (2013). Glycobiome: bacteria and mucus at the epithelial interface. Best Pract Res Clin Gastroenterol.

[8] Willing BP, Dicksved J, Halfvarson J, Andersson AF, Lucio M (2010). A pyrosequencing study in twins shows that gastrointestinal microbial profiles vary with inflammatory bowel disease phenotypes. Gastroenterology.

[9] Buffie CG, Bucci V, Stein RR, McKenney PT, Ling L (2015). Precision microbiome reconstitution restores bile acid mediated resistance to *Clostridium difficile*. Nature.

[10] Nicholson JK, Holmes E, Kinross J, Burcelin R, Gibson G (2012). Host-gut microbiota metabolic interactions. Science.

[11] Sonnenburg JL, Bäckhed F (2016). Diet-microbiota interactions as moderators of human metabolism. Nature.

[12] Lloyd-Price J, Mahurkar A, Rahnavard G, Crabtree J, Orvis J (2017). Strains, functions and dynamics in the expanded human microbiome project. Nature.

[13] Brial F, Le Lay A, Dumas ME, Gauguier D (2018). Implication of gut microbiota metabolites in cardiovascular and metabolic diseases. Cell Mol Life Sci.

[14] Murugesan S, Nirmalkar K, Hoyo-Vadillo C, García-Espitia M, Ramírez-Sánchez D (2018). Gut microbiome production of short-chain fatty acids and obesity in children. Eur J Clin Microbiol Infect Dis.

[15] LeBlanc JG, Chain F, Martín R, Bermúdez-Humarán LG, Courau S (2017). Beneficial effects on host energy metabolism of short-chain fatty acids and vitamins produced by commensal and probiotic bacteria. Microb Cell Fact.

[16] De Vadder F, Kovatcheva-Datchary P, Goncalves D, Vinera J, Zitoun C (2014). Microbiota-generated metabolites promote metabolic benefits via gut-brain neural circuits. Cell.

[17] Bennett BJ, Vallim TQ de A, Wang Z, Shih DM, Meng Y (2013). Trimethylamine-N-oxide, a metabolite associated with atherosclerosis, exhibits Complex genetic and dietary regulation. Cell Metabolism.

[18] Hoyles L, Jiménez-Pranteda ML, Chilloux J, Brial F, Myridakis A (2018). Metabolic retroconversion of trimethylamine N-oxide and the gut microbiota. Microbiome.

[19] Pedersen HK, Gudmundsdottir V, Nielsen HB, Hyotylainen T, Nielsen T (2016). Human gut microbes impact host serum metabolome and insulin sensitivity. Nature.

[20] Parkar SG, Trower TM, Stevenson DE (2013). Fecal microbial metabolism of polyphenols and its effects on human gut microbiota. Anaerobe.

[21] Russell WR, Duncan SH, Scobbie L, Duncan G, Cantlay L (2013). Major phenylpropanoid-derived metabolites in the human gut can arise from microbial fermentation of protein. Mol Nutr Food Res.

[22] Dall’Asta M, Calani L, Tedeschi M, Jechiu L, Brighenti F (2012). Identification of microbial metabolites derived from in vitro fecal fermentation of different polyphenolic food sources. Nutrition.

[23] Caetano BFR, de Moura NA, Almeida APS, Dias MC, Sivieri K (2016). Yacon (*Smallanthus sonchifolius*) as a food supplement: health-promoting benefits of Fructooligosaccharides. Nutrients.

[24] Du W, Xu H, Mei X, Cao X, Gong L (2018). Probiotic bacillus enhance the intestinal epithelial cell barrier and immune function of piglets. Benef Microbes.

[25] Ren Z, Li Y, Liu J, Li H, Li A (2018). *Coreopsis tinctoria* modulates lipid metabolism by decreasing low-density lipoprotein and improving gut microbiota. Cell Physiol Biochem.

[26] Chen D, Yang Z, Chen X, Huang Y, Yin B (2015). Effect of *Lactobacillus rhamnosus* hsryfm 1301 on the gut microbiota and lipid metabolism in rats fed a high-fat diet. J Microbiol Biotechnol.

[27] Uebanso T, Kano S, Yoshimoto A, Naito C, Shimohata T (2017). Effects of consuming xylitol on gut microbiota and lipid metabolism in Mice. Nutrients.

[28] Li TT, Liu YY, Wan XZ, Huang ZR, Liu B (2018). Regulatory efficacy of the polyunsaturated fatty acids from microalgae *Spirulina platensis* on lipid metabolism and gut microbiota in high-fat diet rats. Int J Mol Sci.

[29] Zhou SM, Wang YQ, Jacoby JJ, Jiang YR, Zhang YQ (2017). Effects of medium- and long-chain triacylglycerols on lipid metabolism and gut microbiota composition in C57BL/6J Mice. J Agric Food Chem.

[30] Wan J, Hu S, Jacoby JJ, Liu J, Zhang Y (2017). The impact of dietary sn-2 palmitic triacylglycerols in combination with docosahexaenoic acid or arachidonic acid on lipid metabolism and host faecal microbiota composition in Sprague Dawley rats. Food Funct.

[31] Zhou L, Xiao X, Zhang Q, Zheng J, Li M (2018). Improved glucose and lipid metabolism in the early life of female offspring by maternal dietary genistein is associated with alterations in the gut microbiota. Front Endocrinol.

[32] Ye J, Lv L, Wu W, Li Y, Shi D (2018). Butyrate protects Mice against methionine–choline-deficient diet-induced non-alcoholic steatohepatitis by improving gut barrier function, attenuating inflammation and reducing endotoxin levels. Front Microbiol.

[33] Jin Y, Wu Y, Zeng Z, Jin C, Wu S (2016). From the cover: exposure to oral antibiotics induces gut microbiota dysbiosis associated with lipid metabolism dysfunction and low-grade inflammation in Mice. Toxicol Sci.

[34] Jin YX, Zeng ZY, Wu Y, Zhang SB, Fu ZW (2015). Oral exposure of mice to carbendazim induces hepatic lipid metabolism disorder and gut microbiota dysbiosis. Toxicol Sci.

[35] Yin J, Li Y, Han H, Chen S, Gao J (2018). Melatonin reprogramming of gut microbiota improves lipid dysmetabolism in high-fat diet-fed mice. J Pineal Res.

[36] Araya-Cloutier C, den Besten HMW, Aisyah S, Gruppen H, Vincken J-P (2017). The position of prenylation of isoflavonoids and stilbenoids from legumes (Fabaceae) modulates the antimicrobial activity against Gram positive pathogens. Food Chem.

[37] Espley RV, Butts CA, Laing WA, Martell S, Smith H (2014). Dietary flavonoids from modified apple reduce inflammation markers and modulate gut microbiota in mice. J Nutr.

[38] Masumoto S, Terao A, Yamamoto Y, Mukai T, Miura T (2016). Non-absorbable apple procyanidins prevent obesity associated with gut microbial and metabolomic changes. Sci Rep.

[39] Roberfroid M, Gibson GR, Hoyles L, McCartney AL, Rastall R (2010). Prebiotic effects: metabolic and health benefits. Br J Nutr.

[40] Valcheva R, Dieleman LA (2016). Prebiotics: definition and protective mechanisms. Best Pract Res Clin Gastroenterol.

[41] Wang C, Yue S, Hao Z, Ren G, Lu D (2019). Pubertal exposure to the endocrine disruptor mono-2-ethylhexyl ester at body burden level caused cholesterol imbalance in mice. Environmental Pollution.

[42] Yu Y, Liu Q, Li H, Wen C, He Z (2018). Alterations of the gut microbiome associated with the treatment of hyperuricaemia in male rats. Front Microbiol.

[43] Ijaz MU, Ahmed MI, Zou X, Hussain M, Zhang M (2018). Beef, casein, and soy proteins differentially affect lipid metabolism, triglycerides accumulation and gut microbiota of high-fat diet-fed C57BL/6J Mice. Front Microbiol.

[44] Li Y, Fu X, Ma X, Geng S, Jiang X (2018). Intestinal microbiome-metabolome responses to essential oils in piglets. Front Microbiol.

[45] Alfawaz H, Al-Onazi M, Bukhari SI, Binobead M, Othman N (2018). The independent and combined effects of omega-3 and vitamin B12 in ameliorating propionic acid induced biochemical features in juvenile rats as rodent model of autism. J Mol Neurosci.

[46] Brown EM, Sadarangani M, Finlay BB (2013). The role of the immune system in governing host-microbe interactions in the intestine. Nat Immunol.

[47] Pei R, Martin DA, DiMarco DM, Bolling BW (2017). Evidence for the effects of yogurt on gut health and obesity. Crit Rev Food Sci Nutr.

[48] Hooper LV, Macpherson AJ (2010). Immune adaptations that maintain homeostasis with the intestinal microbiota. Nat Rev Immunol.

[49] Cani PD (2016). Interactions between gut microbes and host cells control gut barrier and metabolism. Int J Obes Suppl.

[50] Muccioli GG, Naslain D, Bäckhed F, Reigstad CS, Lambert DM (2010). The endocannabinoid system links gut microbiota to adipogenesis. Mol Syst Biol.

[51] Cani PD, Plovier H, Van Hul M, Geurts L, Delzenne NM (2016). Endocannabinoids--at the crossroads between the gut microbiota and host metabolism. Nat Rev Endocrinol.

[52] Everard A, Belzer C, Geurts L, Ouwerkerk JP, Druart C (2013). Cross-talk between *Akkermansia muciniphila* and intestinal epithelium controls diet-induced obesity. Proc Natl Acad Sci.

[53] Alhouayek M, Lambert DM, Delzenne NM, Cani PD, Muccioli GG (2011). Increasing endogenous 2-arachidonoylglycerol levels counteracts colitis and related systemic inflammation. FASEB J.

[54] Cani PD, Possemiers S, Van de Wiele T, Guiot Y, Everard A (2009). Changes in gut microbiota control inflammation in obese mice through a mechanism involving GLP-2-driven improvement of gut permeability. Gut.

[55] Birchenough G, Schroeder BO, Bäckhed F, Hansson GC (2019). Dietary destabilisation of the balance between the microbiota and the colonic mucus barrier. Gut Microbes.

[56] Kobyliak N, Conte C, Cammarota G, Haley AP, Styriak I (2016). Probiotics in prevention and treatment of obesity: a critical view. Nutr Metab.

[57] Roselli M, Devirgiliis C, Zinno P, Guantario B, Finamore A (2017). Impact of supplementation with a food-derived microbial community on obesity-associated inflammation and gut microbiota composition. Genes Nutr.

[58] Festi D, Schiumerini R, Eusebi LH, Marasco G, Taddia M (2014). Gut microbiota and metabolic syndrome. World J Gastroenterol.

[59] Roxas JL, Vedantam G, Viswanathan VK (2019). Epithelial maturity influences EPEC-induced desmosomal alterations. Gut Microbes.

[60] Bai J, Hu Y, Bruner DW (2019). Composition of gut microbiota and its association with body mass index and lifestyle factors in a cohort of 7-18 years old children from the American Gut Project. Pediatr Obes.

[61] Priyadarshini M, Wicksteed B, Schiltz GE, Gilchrist A, Layden BT (2016). SCFA receptors in pancreatic β cells: novel diabetes targets?. Trends Endocrinol Metab.

[62] Chambers ES, Morrison DJ, Frost G (2015). Control of appetite and energy intake by SCFA: what are the potential underlying mechanisms?. Proc Nutr Soc.

[63] Clemente-Postigo M, Oliva-Olivera W, Coin-Aragüez L, Ramos-Molina B, Giraldez-Perez RM (2019). Metabolic endotoxemia promotes adipose dysfunction and inflammation in human obesity. Am J Physiol Endocrinol Metab.

[64] Dalby MJ, Aviello G, Ross AW, Walker AW, Barrett P (2018). Diet induced obesity is independent of metabolic endotoxemia and TLR4 signalling, but markedly increases hypothalamic expression of the acute phase protein, SerpinA3N. Sci Rep.

[65] Wang Y, Fei Y, Liu L, Xiao Y, Pang Y (2018). *Polygonatum odoratum* polysaccharides modulate gut microbiota and mitigate experimentally induced obesity in rats. Int J Mol Sci.

[66] Moreno-Indias I, Sánchez-Alcoholado L, Pérez-Martínez P, Andrés-Lacueva C, Cardona F (2016). Red wine polyphenols modulate fecal microbiota and reduce markers of the metabolic syndrome in obese patients. Food Funct.

[67] Mehrpouya-Bahrami P, Chitrala KN, Ganewatta MS, Tang C, Murphy EA (2017). Blockade of CB1 cannabinoid receptor alters gut microbiota and attenuates inflammation and diet-induced obesity. Sci Rep.

[68] Qin J, Li Y, Cai Z, Li S, Zhu J (2012). A metagenome-wide association study of gut microbiota in type 2 diabetes. Nature.

[69] Buffie CG, Bucci V, Stein RR, McKenney PT, Ling L (2015). Precision microbiome reconstitution restores bile acid mediated resistance to *Clostridium difficile*. Nature.

[70] Reijnders D, Goossens GH, Hermes GDA, Neis EPJG, van der Beek CM (2016). Effects of gut microbiota manipulation by antibiotics on host metabolism in obese humans: a randomized double-blind placebo-controlled trial. Cell Metabolism.

[71] Henagan TM, Stefanska B, Fang Z, Navard AM, Ye J (2015). Sodium butyrate epigenetically modulates high-fat diet-induced skeletal muscle mitochondrial adaptation, obesity and insulin resistance through nucleosome positioning. Br J Pharmacol.

[72] Lin YC, Chen YT, Hsieh HH, Chen MJ (2016). Effect of *Lactobacillus mali* APS1 and *L. kefiranofaciens* M1 on obesity and glucose homeostasis in diet-induced obese mice. J Funct Foods.

[73] Li C, Li X, Han H, Cui H, Peng M (2016). Effect of probiotics on metabolic profiles in type 2 diabetes mellitus: a meta-analysis of randomized, controlled trials. Medicine.

[74] Laurans L, Venteclef N, Haddad Y, Chajadine M, Alzaid F (2018). Genetic deficiency of indoleamine 2,3-dioxygenase promotes gut microbiota-mediated metabolic health. Nat Med.

[75] Collins HL, Drazul-Schrader D, Sulpizio AC, Koster PD, Williamson Y (2016). L-Carnitine intake and high trimethylamine N-oxide plasma levels correlate with low aortic lesions in ApoE(-/-) transgenic mice expressing CETP. Atherosclerosis.

[76] Griffin JL, Wang X, Stanley E (2015). Does our gut microbiome predict cardiovascular risk? A review of the evidence from metabolomics. Circ Cardiovasc Genet.

